# Comparative assessment of thawing methods for frozen rooster sperm

**DOI:** 10.3389/fvets.2025.1562053

**Published:** 2025-04-04

**Authors:** Mustafa Yigit Nizam, Murat Selçuk, Murat Kirikkulak

**Affiliations:** ^1^Department of Reproduction and Artificial Insemination, Faculty of Veterinary Medicine, Dokuz Eylül University, Izmir, Türkiye; ^2^Department of Reproduction and Artificial Insemination, Faculty of Veterinary Medicine, Ondokuz Mayis University, Samsun, Türkiye; ^3^Department of Reproduction and Artificial Insemination, Faculty of Veterinary Medicine, Afyon Kocatepe University, Afyonkarahisar, Türkiye

**Keywords:** COMET, cryopreservation, DNA integrity, dry thawing, poultry

## Abstract

**Introduction:**

Cryopreservation, widely used in commercial poultry breeding, often reduces sperm motility, viability, and DNA integrity due to cryopreservation-induced damage. This study evaluates the effects of water bath and dry thawing systems on the post-thaw quality of rooster spermatozoa, addressing these challenges and exploring methods to enhance sperm quality.

**Methods:**

The study compared the performance of water bath and dry thawing systems, both operated at 37°C for 30 s. Post-thaw assessments included sperm motility, morphology, kinematic characteristics, and DNA integrity. Key parameters such as total motility, progressive motility, curvilinear velocity (VCL), average path velocity (VAP), straight-line velocity (VSL), viability, morphological abnormalities, and DNA damage metrics were analyzed.

**Results:**

The dry thawing system significantly improved sperm quality compared to the water bath method. Total motility and progressive motility were higher in the dry thawing system (82.38 and 33.18%, respectively) compared to the water bath method (68.14 and 21.20%). Kinematic parameters, including VCL (79.41 vs. 66.49 μm/s), VAP (47.52 vs. 37.42 μm/s), and VSL (27.18 vs. 21.59 μm/s), were superior in the dry thawing system. Viability improved (82.2 vs. 73.7%), while morphological abnormalities were reduced (23.9 vs. 35.8%). DNA integrity metrics, such as Tail DNA (%; 77.37 vs. 81.11%) and Olive Tail Moment (15.28 vs. 16.93), also showed reduced damage.

**Discussion:**

The dry thawing system offers significant operational advantages, including portability, contamination-free operation, and consistent temperature maintenance, making it ideal for on-site applications. These features, combined with its ability to enhance sperm quality, highlight the dry thawing system as an effective alternative for poultry breeding. Its adoption could improve artificial insemination outcomes and address challenges associated with cryopreservation-induced damage during thawing.

## Introduction

Cryopreservation of avian semen has been widely utilized to establish effective artificial insemination techniques for commercial breeding programs (1). Nevertheless, the fertility potential of thawed semen remains suboptimal due to the reduced motility and viability of rooster sperm following the freeze-thaw process (2). This challenge primarily arises from various types of damage incurred during cryopreservation, including mechanical, biochemical, and ultra-structural changes (3), which impair sperm quality and the fertility potential of thawed samples. Furthermore, rooster sperm are particularly prone to cryo-damage (4) because their plasma membranes contain a higher proportion of polyunsaturated fatty acids (5) and their cytoplasm has lower levels of scavenging enzymes (6) compared to other species (7). Thus, a refined approach is essential to mitigate the detrimental effects of cryo-injuries (8). Over the past 24 years, antioxidants have been routinely employed as a protective measure against cryo-damage (9, 10). However, this strategy has proven insufficient in fully preventing cryopreservation-induced damage, likely due to the limited efficacy of antioxidants and their potential conversion into toxic by-products that may disrupt key cellular functions (11).

The structural integrity of spermatozoa membranes is susceptible to variations in freezing and thawing temperatures. Following the thawing process, spermatozoa exhibit greater susceptibility to damage compared to their fresh counterparts, largely due to the physical and biochemical stresses associated with the freeze-thaw cycle. The post-thaw viability of spermatozoa is significantly influenced by the specific thawing protocol employed, including factors such as the choice of thawing medium (e.g., water or air) and the temperature conditions used during the procedure (12). For optimal results, spermatozoa must be thawed at the fastest possible rate, as rapid thawing has been demonstrated to improve motility significantly (13). Numerous studies have focused on determining the ideal thawing temperature to maximize the percentage of viable spermatozoa while ensuring the highest possible recovery of functional cells. These findings underscore the critical role of precise temperature management in achieving optimal post-thaw sperm quality (12–15).

Such practices reflect ongoing efforts to refine thawing protocols for improved reproductive outcomes in poultry species. Several studies have examined the thawing process of rooster spermatozoa, focusing on a range of temperatures and durations to optimize post-thaw sperm quality. These investigations have particularly emphasized the effects of varying thawing times and temperatures on the structural and functional integrity of spermatozoa (16, 17). Among the methods explored in recent publications, avian sperm is most commonly thawed in a water bath maintained at 37°C for 30 s, as this temperature is considered standard for achieving favorable results in terms of motility, viability, and overall post-thaw performance (18).

The dry thawing system offers several advantages over the conventional water bath method for sperm thawing, addressing key challenges such as the risk of contamination from water mixing and the difficulty of maintaining consistent water temperatures, particularly in colder environments. Its portability and ease of use make it especially suitable for on-site applications in farms and barns, where its self-contained heating mechanism ensures the required temperature of 37°C for up to 10 min. Additionally, the system eliminates the need for drying straws post-thawing and features specialized slots for straws of varying sizes and artificial insemination catheters, streamlining the preparation process (19).

Building on these benefits, this study aimed to compare the effects of water bath and dry thawing systems, both operating at 37°C for 30 s, on critical sperm quality parameters, including motility, morphology, kinematic characteristics, viability, abnormal spermatozoa, and DNA integrity. DNA damage was meticulously assessed using the COMET assay, ensuring a robust evaluation of the impact of these thawing methods on genetic material integrity.

## Materials and methods

This study was conducted in compliance with the ethical standards approved by the Poultry Research Institute Ethics Committee (Approval No. 2020/10), operating under the Republic of Türkiye Ministry of Agriculture and Forestry. Within this ethical framework, twenty 49-week-old Plymouth Rock roosters were housed in individual cages under controlled conditions, with a lighting schedule of 16 h of light and 8 h of darkness. Semen was collected once from each rooster using the dorso-abdominal massage (20). Only samples with an initial motility of 90% or higher were included in the study.

To minimize individual variability, semen from all roosters was pooled into a single collection, ensuring a uniform sample for further processing. The pooled semen was then diluted with Beltsville Poultry Semen Extender (BPSE) supplemented with 5% glycerol as a cryoprotectant. After dilution, the semen was loaded into 0.25 mL straws and subjected to equilibration at 4°C for 2 h. Following equilibration, the straws were frozen in liquid nitrogen vapor and subsequently immersed in liquid nitrogen for long-term storage.

### Thawing procedures

**Figure 1:**
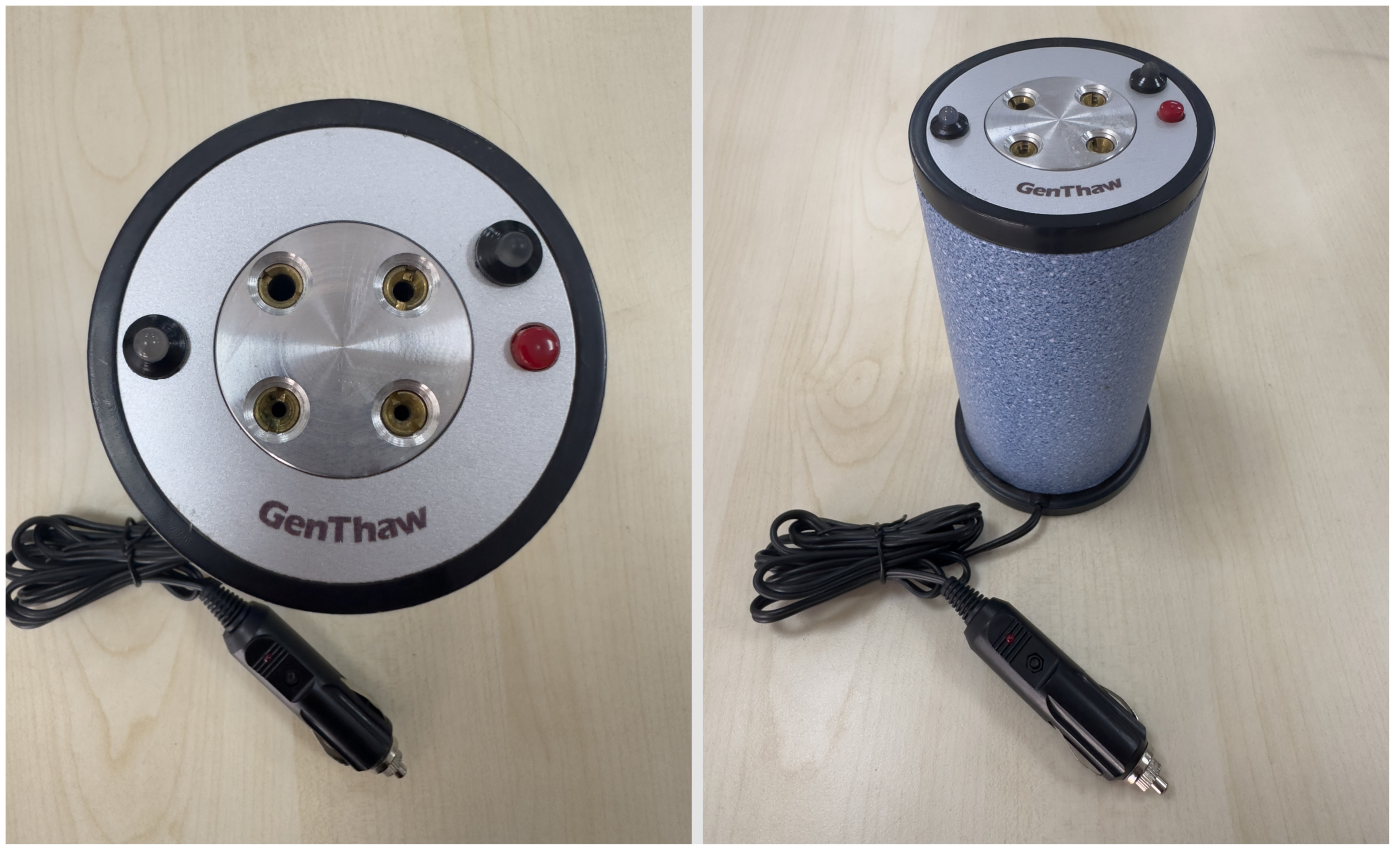
Dry thawing system.

This study compared two distinct thawing methods for rooster semen stored in 0.25 mL straws:

1. Water bath: Straws were thawed in a water bath maintained at 37°C for 30 s.
2. Dry thawing system: Straws were thawed using an innovative, portable dry thawing device set to 37°C for 30 s (Figure 1).

The dry thawing system is equipped with four designated slots on its top surface, each tailored for specific uses: one for catheter warming prior to artificial insemination (AI), one for 0.50 mL straws, and two for 0.25 mL straws. The device operates using a 12–13.6 V power source, making it portable and compatible with a vehicle's lighter socket. It is capable of maintaining the target temperature for ~10 min, ensuring consistency throughout the thawing process.

A total of 10 straws were processed for each thawing method.

### Sperm motion parameters

The assessment of total motility and progressive motility was performed using a computer-assisted sperm analysis system (CASA; Sperm Class Analyzer^®^, version 6.3.0.59, Microptic, Barcelona, Spain). A pre-warmed microscope stage maintained at 37°C was utilized for slide placement during analysis. For each sample, a minimum of 500 spermatozoa from at least five distinct microscopic fields were evaluated. To determine the kinematic properties of sperm movement, parameters such as straight-line velocity (VSL; μm/s), curvilinear velocity (VCL; μm/s), average path velocity (VAP; μm/s), amplitude of lateral head displacement (ALH; μm), linearity (LIN; calculated as VSL/VCL × 100), wobble (WOB; calculated as VAP/VCL × 100), straightness (STR; calculated as VSL/VAP × 100), and beat-cross frequency (BCF; Hz) were measured using the integrated software.

### Sperm morphology

Sperm morphology was assessed using Hancock's solution, following the method described by Najafi et al. (21). The proportions of abnormalities in the head, mid-piece, tail, and total spermatozoa were quantified as percentages (%). A small volume of semen was combined with Hancock's solution and spread onto a microscope slide. Anomalies in the spermatozoa were examined under a phase-contrast microscope (Eclipse Ci-L, Nikon, Japan) at 100 × magnification, with 200 spermatozoa analyzed per slide.

### Sperm viability

Sperm viability was evaluated using the eosin-nigrosine staining technique, as described by Raseona et al. (22). After staining, the slides were air-dried, covered with a coverslip, and examined under a phase-contrast microscope (Eclipse Ci-L, Nikon, Japan) at 60 × magnification. The proportion of live and dead spermatozoa was determined by analyzing 200 cells per slide, and the viability rate was calculated as a percentage.

### DNA integrity: comet assay

Rooster semen was thawed using two distinct procedures. Post-thaw, the semen samples were transferred to Eppendorf tubes, diluted at a 1:1 ratio with Phosphate-Buffered Saline (PBS) devoid of Ca^+2^ and Mg^+2^, and subjected to centrifugation at +4°C for 10 min at 800 rpm. The supernatant was carefully discarded, and the spermatozoa were reconstituted in PBS. This washing process was repeated, and following the removal of the supernatant after the second centrifugation, the spermatozoa were diluted again at a 1:1 ratio with PBS (23, 24).

A total of 120 μL of 0.75% Low-Melting Agarose (LMA) gel, prepared in PBS, was applied onto sandblasted slides and evenly spread across the surface. The slides were then left to dry at room temperature, forming the first agarose layer. Subsequently, 10 μL of semen diluted with PBS was combined with 90 μL of 1% LMA gel in an Eppendorf tube maintained at 37°C. The resulting 100 μL mixture was evenly distributed over the initial agarose layer, covered with a 24 × 60 mm coverslip, and placed on an ice pack to allow solidification. Once solidified, the coverslips were gently removed, completing the preparation of the slides (25, 26).

The lysis solution facilitates the disruption of cellular and nuclear membranes, enabling the release of DNA strands embedded within the agarose gel. Following the embedding of spermatozoa into the agarose layer on the prepared slides, the slides were placed in a Coplin jar and incubated at +4°C for 1 h in Comet Assay Lysis Solution (R&D Systems, Catalog No: 4250-050-01), which contained high concentrations of salt, detergent, and 1% Triton X-100. After the initial incubation, 1 mL of Dithiothreitol (DDT) was introduced into the lysis solution, and the slides were incubated for an additional hour at +4°C. Subsequently, 0.5 mL of Proteinase K was added to the Coplin jar, and the slides were transferred to an incubator and maintained at +37°C overnight (25, 26).

The samples, prepared with modifications based on the method outlined by Shanmugam et al. (27), were incubated for 20 min in a freshly prepared, cooled electrophoresis buffer solution (TAE, pH 7.3) within an electrophoresis tank to facilitate the separation of DNA strands prior to electrophoresis. Following the incubation of spermatozoa embedded in the agarose layer, the samples were subjected to electrophoresis in the same buffer solution under an electrical field of 20 volts and 30 mA for 15 min. Upon completion of electrophoresis, the slides were rinsed with a freshly prepared Tris Buffer Solution (0.4 M Tris HCl, pH 7.5) to eliminate residual electrophoresis solution from the samples.

Following the completion of the neutralization process, the DNA was stained with the fluorescent dye ethidium bromide at a concentration of 5 μg/mL. To achieve this, a drop of ethidium bromide was applied to the samples, which were subsequently covered with a 24 × 60 mm coverslip and analyzed within 4 h, as outlined by Gliozzi et al. (28).

Ethidium bromide-stained samples were analyzed at 400 × magnification using a phase-contrast microscope equipped with a fluorescent attachment (Olympus CX-31). A total of 100 comet images from each group were evaluated using TriTek Comet Score™ Freeware v1.5. All analyses were conducted under dim light conditions to minimize the risk of additional DNA damage (29). DNA damage was quantified based on the parameters of Tail DNA (%), Tail Length (μm), Comet Length (μm), and Olive Tail Moment, recorded during the evaluation.

### Statistical analysis

All data in the study were subjected to homogeneity test of variances. Considering the number of groups, the data were analyzed using Independent Sample *T*-Test. Statistically significant differences were considered at *p* < 0.001 level. All results were presented as mean ± standard deviation. All statistical analyses were performed using IBM SPSS version 29 (Chicago, USA).

## Results

### Sperm motion parameters

The motility and kinematic parameters of spermatozoa showed significant differences between the two thawing methods (Table 1). Total motility was significantly higher in the dry thawing system (82.38 ± 5.66) compared to the water bath method (68.14 ± 7.06; *P* < 0.001). Progressive motility, including rapid and medium progressive sperm, also exhibited notable improvement in the dry thawing system (33.18 ± 8.47) compared to the water bath method (21.20 ± 7.11; *P* < 0.001).

**Table 1:** Sperm motion parameters after thawing (mean ± standard error).

**Parameters**	**Dry system**	**Water bath**	***P*-value**
Motility (%)	82.38 ± 5.66	68.14 ± 7.06	^*^
Progressive motility (%)	33.18 ± 8.47	21.20 ± 7.11	^*^
VCL (μm/s)	79.41 ± 11.12	66.49 ± 12.58	^*^
VAP (μm/s)	47.52 ± 8.13	37.42 ± 6.46	^*^
VSL (μm/s)	27.18 ± 4.72	21.59 ± 3.20	^*^
STR (%)	50.93 ± 1.79	50.58 ± 2.15	NS
LIN (%)	32.49 ± 2.14	31.31 ± 2.62	NS
WOB (%)	58.53 ± 3.80	53.77 ± 3.36	^*^
ALH (μm)	2.40 ± 0.28	2.07 ± 0.33	^*^
BCF (Hz)	5.63 ± 1.11	3.81 ± 0.49	^*^

NS, Not significant.

^*^P < 0.001.

In terms of kinematic parameters, the dry thawing system demonstrated superior values compared to the water bath thawing system for VCL (79.41 ± 11.12, 66.49 ± 12.58, respectively; *P* < 0.001), average path velocity (VAP: 47.52 ± 8.13, 37.42 ± 6.46, respectively; *P* < 0.001), and straight-line velocity (VSL: 27.18 ± 4.72, 21.59 ± 3.20, respectively; *P* < 0.001). The amplitude of lateral head displacement (ALH) and beat-cross frequency (BCF) were also significantly higher in the dry thawing system (*P* < 0.001). However, no significant differences were observed in straightness (STR) and linearity (LIN) between the two methods (*P* > 0.05).

### Sperm viability and morphology

Sperm viability was significantly improved in the dry thawing system, with a mean value of 82.2 ± 1.68 compared to 73.7 ± 1.49 observed in the water bath method (*P* < 0.01). Morphological assessments also revealed notable differences between the two methods (Table 2). Specifically, the dry thawing system demonstrated fewer abnormalities in the head (4.0 ± 0.81 compared to 7.8 ± 0.63 in the water bath method; *P* < 0.01), midpiece (4.0 ± 0.66 compared to 7.0 ± 0.66 in the water bath method; *P* < 0.01), and tail (15.9 ± 0.87 compared to 21.0 ± 0.94 in the water bath method; *P* < 0.01) regions. Total morphological abnormalities were also significantly lower in the dry thawing system (23.9 ± 1.66) compared to 35.8 ± 1.13 in the water bath method (*P* < 0.01).

**Table 2:** Post thaw sperm viability and morphology parameters (mean ± standard error).

	**Parameters**	**Dry system**	**Water bath**	***P*-value**
	**Viability**	82.2 ± 1.68	73.7 ± 1.49	^*^
**Morphology**	Head (%)	4 ± 0.81	7.8 ± 0.63	^*^
	Midpiece (%)	4 ± 0.66	7 ± 0.66	^*^
	Tail (%)	15.9 ± 0.87	21 ± 0.94	^*^
	Total (%)	23.9 ± 1.66	35.8 ± 1.13	^*^

^*^P < 0.01.

### COMET assay results

The COMET assay indicated a reduction in DNA damage with the dry thawing system (Table 3). Although tail length did not differ significantly between dry thawing and water bath thawing (19.22 ± 0.74 and 20.15 ± 1.43, respectively; *P* = 0.085), the Tail DNA (%) was significantly lower in the dry thawing system (77.37 ± 2.29) compared to the water bath method (81.11 ± 4.55; *P* = 0.037). Olive tail moment, a marker of DNA fragmentation, was also significantly reduced in the dry thawing system (15.28 ± 0.65) compared to the water bath (16.93 ± 1.43; *P* = 0.006).

**Table 3:** Post-thaw COMET results (mean ± standard error).

**Parameters**	**Dry thawing**	**Water bath**	***P*-value**
Tail length (px)	19.22 ± 0.74	20.15 ± 1.43	NS
Tail DNA (%)	77.37 ± 2.29	81.11 ± 4.55	^*^
Olive tail moment	15.28 ± 0.65	16.93 ± 1.43	^*^

NS, Not Significant.

^*^P < 0.05.

## Discussion

The impact of thawing methods on sperm motility and progressive motility has been widely investigated, revealing notable differences among various approaches. Masoudi et al. (30), who employed a water bath thawing method, observed a lower overall motility rate, despite reporting relatively higher progressive motility. Similarly, Najafi et al. (31) documented reduced values for both motility and progressive motility using the same method, highlighting potential limitations associated with water-based thawing techniques. In studies by Najafi et al. (32, 33), which also relied on water bath thawing, motility rates were comparable to those of the water bath group in this study, yet progressive motility exhibited some variability. While one study aligned more closely with dry thawing results, the other presented noticeably lower values. Additionally, Salehi et al. (34), who also applied water bath thawing, reported relatively higher progressive motility, though overall motility remained below that achieved with dry thawing. The present findings suggest that the dry thawing system fosters a more stable thawing environment, resulting in consistently higher motility and progressive motility compared to water bath thawing. One key factor contributing to this outcome is the controlled thermal transition provided by dry thawing, which prevents abrupt temperature fluctuations that may compromise sperm structure and function. Furthermore, unlike water bath thawing, dry thawing eliminates direct water contact, reducing the risk of potential toxic effects while aiding in the preservation of membrane integrity. Another advantage of the dry thawing system is its enclosed design, which promotes even heat distribution and minimizes thermal loss. This ensures a gradual and uniform temperature increase, preventing localized overheating or cooling, both of which can disrupt sperm kinetics. While progressive motility may, in some cases, be maintained in water bath thawing, the overall trend indicates that sperm motility is more effectively preserved with dry thawing, reinforcing its practical benefits for post-thaw sperm viability. Moving forward, further refinements in dry thawing protocols could enhance its effectiveness in maintaining optimal sperm motility and progressive motility, ensuring better outcomes for assisted reproduction techniques.

In this study, the dry thawing method demonstrated superior kinematic performance, further emphasizing the crucial role of thawing methodology in preserving sperm motility and function. Notably, curvilinear velocity (VCL) was significantly higher in the dry thawing system compared to the water bath method, suggesting that this technique more effectively maintains dynamic sperm movement, which is essential for fertilization. Similarly, improvements in average path velocity (VAP) and straight-line velocity (VSL) further highlight the capacity of dry thawing to enhance sperm trajectory and forward progression. Comparison with previous studies further substantiates the advantages of dry thawing. Feyzi et al. (35) and Najafi et al. (32) reported lower VCL values with water bath thawing, reinforcing the efficacy of dry thawing in maintaining sperm motility. Similarly, although Salehi et al. (33) observed a higher VAP than both thawing methods in this study, variations in methodology such as extender composition or post-thaw incubation conditions may have influenced the discrepancies. While VSL values in dry thawing were superior to those obtained through water bath thawing, they remained below the levels reported by Feyzi et al. (35) and Masoudi et al. (30), suggesting that further optimization may enhance this parameter. In terms of additional kinematic properties, linearity (LIN) and straightness (STR) values exhibited minimal differences between thawing methods, yet remained lower than those reported in previous studies, suggesting deviations in sperm trajectory. Conversely, amplitude of lateral head displacement (ALH) and beat-cross frequency (BCF) were slightly higher in the dry thawing system, indicating enhanced motility regulation. However, Najafi et al. (31, 32) reported considerably higher BCF values, reflecting potential methodological variations across different studies. Collectively, these findings underscore the efficacy of dry thawing in preserving key kinematic parameters, particularly VCL, VAP, and VSL, while also identifying areas requiring further refinement specifically, improvements in linearity and directional movement. Future research should explore modifications in thawing conditions, such as fine-tuning temperature gradients and optimizing post-thaw incubation strategies, to achieve greater kinematic stability and overall sperm functionality.

The dry thawing method exhibited the highest sperm viability rate compared to the water bath method and previously reported values in the literature, highlighting its effectiveness in preserving sperm cell integrity and functionality. Comparison with previous studies further supports these advantages. Feyzi et al. (35) and Najafi et al. (32) reported viability rates similar to those observed with the water bath method in this study, yet lower than those achieved with dry thawing. Likewise, Najafi et al. (31) found viability rates consistent with water bath thawing, reinforcing the reliability of conventional thawing methods. In contrast, Masoudi et al. (30) documented considerably lower viability rates with water bath thawing, emphasizing the protective effects of dry thawing in reducing cell damage. Similarly, Salehi et al. (33) reported the lowest viability rates among the reviewed studies, further demonstrating the limitations of less optimized thawing protocols. These comparisons highlight the ability of the dry thawing system to reduce sperm deterioration during the thawing process, likely due to its controlled temperature regulation and reduced exposure to thermal fluctuations. Overall, these findings confirm the effectiveness of the dry thawing system in preserving sperm viability and maintaining cell membrane integrity. Its superior performance suggests that it could be a reliable and practical method for post-thaw sperm optimization. Future research should focus on its role in minimizing cryo-damage, evaluating its impact on fertilization rates, and determining its scalability for widespread use in artificial insemination programs.

The dry thawing system resulted in a notably lower total sperm morphology abnormality rate compared to the water bath method, further emphasizing its superiority in preserving sperm structural integrity and minimizing cryo-induced damage. Nevertheless, despite its advantages, the abnormality rate remains higher than those reported in some previous studies. This discrepancy could be attributed to variations in cryopreservation protocols, semen handling techniques, and extender compositions, all of which play a crucial role in post-thaw sperm integrity. This discrepancy may stem from differences in cryopreservation protocols, semen handling techniques, or the composition of extenders used during freezing and thawing. Additionally, variations in species-specific sperm characteristics and storage conditions could also contribute to these differences. Despite this variation, the dry thawing system demonstrated a clear advantage over the water bath method in minimizing sperm abnormalities. These findings suggest that further refinement of thawing protocols and optimization of extender formulations may help achieve abnormality rates closer to those reported in the literature. Future research should focus on identifying the underlying factors contributing to sperm deformities and determining whether additional modifications to the dry thawing system can further enhance post-thaw sperm morphology.

The results of this study highlight the notable advantages of the dry thawing system over the water bath method in minimizing DNA damage, as evidenced by the COMET assay. Specifically, the Tail DNA (%) for the dry thawing system was significantly lower than that of the water bath method. Similarly, the Tail Moment, another key indicator of DNA integrity, was markedly reduced in the dry thawing system compared to the water bath. While the Tail Length showed no statistically significant difference, the overall trend suggests better preservation of DNA structure with the dry thawing system. When compared to Gliozzi et al. (29), who reported Tail DNA (%) and Tail Moment values, both thawing methods in this study exhibited higher levels of DNA damage. This discrepancy may stem from differences in experimental conditions, sperm handling, or extender compositions. In contrast, Nizam and Selcuk (34) observed substantially lower Tail DNA (%) and Tail Moment values using the water bath method. These variations highlight the potential influence of methodological factors, including temperature gradients and the duration of exposure during thawing, on DNA integrity. The findings from this study emphasize the importance of selecting appropriate thawing techniques to mitigate cryopreservation-induced DNA damage. The dry thawing system's effectiveness in reducing both Tail DNA (%) and Tail Moment suggests that it may help limit cryo-induced structural disruptions. However, the relatively high Tail Length observed across all methods may indicate residual damage inherent to the cryopreservation process, further emphasizing the need for complementary strategies such as antioxidant-enriched extenders or optimized freezing protocols. In comparison to the broader literature, the dry thawing system appears to be a viable alternative to conventional water bath methods, offering a more balanced approach to preserving DNA integrity while maintaining operational simplicity. Future studies should further investigate the mechanisms underlying this method's protective effects, particularly its potential role in reducing oxidative stress and preventing further DNA fragmentation.

## Conclusion

This study highlights the clear advantages of the dry thawing system over the traditional water bath method in preserving the post-thaw quality of rooster spermatozoa. The dry thawing system demonstrated superior performance across various parameters, including total motility, progressive motility, sperm kinematics, morphology, and DNA integrity. Its ability to maintain a consistent and controlled temperature during thawing likely reduces stress on spermatozoa, contributing to the higher motility and progressive motility observed. Additionally, the dry system eliminates the need for water, minimizing contamination risks, which is particularly beneficial in field settings like farms or barns. Its portability and ease of use further enhance its practicality, making it especially valuable in field conditions where operational simplicity and reliability are critical.

The observed differences in sperm quality may also result from the reduced variability introduced by the dry thawing process. By avoiding water-based methods, the system appears to better preserve the structural and functional integrity of spermatozoa, underscoring its effectiveness in maintaining sperm quality. These findings make the dry thawing system a practical and efficient alternative for artificial insemination practices in poultry breeding.

Future research should explore the scalability of the dry thawing system and its direct impact on fertility outcomes. Additionally, investigating the compatibility of this method with diverse extenders and thawing protocols could enhance its applicability and effectiveness in commercial poultry breeding programs.

## Data Availability

The original contributions presented in the study are included in the article/supplementary material, further inquiries can be directed to the corresponding author.
